# A novel patient-derived immortalised cell line of myxofibrosarcoma: a tool for preclinical drugs testing and the generation of near-patient models

**DOI:** 10.1186/s12885-023-11658-9

**Published:** 2023-12-06

**Authors:** Ania Naila Guerrieri, Chiara Bellotti, Marianna Penzo, Marta Columbaro, Micaela Pannella, Alessandro De Vita, Marco Gambarotti, Laura Mercatali, Roberta Laranga, Barbara Dozza, Silvia Vanni, Serena Corsini, Tommaso Frisoni, Giacomo Miserocchi, Toni Ibrahim, Enrico Lucarelli

**Affiliations:** 1 Osteoncology, Bone and Soft Tissue Sarcomas and Innovative Therapies, IRCCS Istituto Ortopedico Rizzoli, Via Di Barbiano 1/10, 40136 Bologna, Italy; 2 Department of Medical and Surgical Sciences and Center for Applied Biomedical Research (CRBA), Alma Mater Studiorum-University of Bologna, 40138 Bologna, Italy; 3 Electron Microscopy Platform, IRCCS Istituto Ortopedico Rizzoli, 40136 Bologna, Italy; 4 Preclinic and Osteoncology Unit, Bioscience Laboratory, IRCCS Istituto Romagnolo Per Lo Studio Dei Tumori (IRST) “Dino Amadori”, 47014 Meldola, Italy; 5 Department of Pathology, IRCCS Istituto Ortopedico Rizzoli, Via Di Barbiano 1/10, 40136 Bologna, Italy; 6 3rd Orthopaedic and Traumatologic Clinic prevalently Oncologic, IRCCS Istituto Ortopedico Rizzoli, Bologna IT, Via Pupilli 1, Bologna, 40136 Italy; 7 Department of Biomedical and Neuromotor Sciences (DIBINEM), Alma Mater Studiorum-University of Bologna, Via Di Barbiano 1/10, 40136 Bologna, Italy; 8 Department of Rare Skeletal Disorders, IRCCS Istituto Ortopedico Rizzoli, Via Di Barbiano 1/10, 40136 Bologna, Italy

**Keywords:** Myxofibrosarcoma, Soft Tissue Sarcoma, Preclinical models, 3D cell culture, Chorioallantoic membrane (CAM)

## Abstract

**Background:**

Myxofibrosarcoma is a rare malignant soft tissue sarcoma characterised by multiple local recurrence and can become of higher grade with each recurrence. Consequently, myxofibrosarcoma represents a burden for patients, a challenge for clinicians, and an interesting disease to study tumour progression. Currently, few myxofibrosarcoma preclinical models are available.

**Methods:**

In this paper, we present a spontaneously immortalised myxofibrosarcoma patient-derived cell line (MF-R 3). We performed phenotypic characterization through multiple biological assays and analyses: proliferation, clonogenic potential, anchorage-independent growth and colony formation, migration, invasion, AgNOR staining, and ultrastructural evaluation.

**Results:**

MF-R 3 cells match morphologic and phenotypic characteristics of the original tumour as 2D cultures, 3D aggregates, and on the chorioallantoic membrane of chick embryos. Overall results show a clear neoplastic potential of this cell line. Finally, we tested MF-R 3 sensitivity to anthracyclines in 2D and 3D conditions finding a good response to these drugs.

**Conclusions:**

In conclusion, we established a novel patient-derived myxofibrosarcoma cell line that, together with the few others available, could serve as an important model for studying the molecular pathogenesis of myxofibrosarcoma and for testing new drugs and therapeutic strategies in diverse experimental settings.

## Introduction

Myxofibrosarcoma (MFS) is one of the most common sarcomas in humans aged 50 and above. In the latest WHO classification, MFS is defined as a malignant fibroblastic neoplasm with variably myxoid stroma, pleomorphism, and a distinctive curvilinear vascular pattern [1]. MFS typically arises as a painless and slow-growing mass with infiltrative margins affecting the extremities [2, 3]. Histologically, MFS is characterised by atypical spindle cells immersed in a gelatinous myxoid matrix with a tendency of multinodular and lobular growth and with curvilinear vessels [4–9]. To date, no specific immunohistochemical markers have been attributed to MFS. However, Goyer *et al.* found a strong positivity for tumour endothelial marker 1 (TEM1) that was not expressed in normal peri-tumoural tissues [10]. At the molecular level, several studies identified karyotypic alterations, gene mutations, and the overexpression and/or mRNA up-regulation of specific genes [1, 2, 11, 12], while others explored the epigenetic, proteomic, metabolomic, and transcriptomic profiles of MFS [8, 13].

Currently, the gold standard treatment of localized MFS is surgery with free margins from neoplastic infiltration, alone or in combination with (neo) adjuvant radio- or chemotherapy [14, 15]. Although surgical negative margins are difficult to obtain because of the intrinsic infiltrative nature of MFS, even if the chance of tumour recurrence is high, positive margins are not correlated with inauspicious prognosis [16]. The major issue is that after several relapses and surgical interventions, patients may undergo amputation [17, 18]. In addition to or before surgery, a chemotherapeutic treatment based on the alone or combined administration of doxorubicin and ifosfamide is mainly used as a palliative intervention due to the lack of consistent data obtained by clinical studies [15].

These clinical challenges make it necessary to deepen the knowledge about MFS neoplastic progression and response to conventional or innovative treatments. Thus far, several studies have been published using in vitro models consisting of cell lines derived from tumour samples that have been successfully used for drug sensitivity experiments [5–7, 12, 19–22]. Unfortunately, the great heterogeneity of MFS is reflected by the different biological behaviour and drug sensitivity displayed by diverse cellular clones isolated from the same patient’s material [12, 23, 24]. Recently, some research groups generated 3D models, using the above-mentioned cell lines grown in ultra-low attachment plates or seeded on collagen scaffolds [19, 21, 23–25]. Additionally, several in vivo animal models have been set up with good engraftment percentages [4, 7, 12, 26].

Primary cell lines still represent fundamental tools to understand the molecular pathogenesis of this sarcoma and to screen the most promising drugs and innovative treatments. Here we describe a novel spontaneously immortalised MFS-derived cell line that we named MF-R 3. We performed an in vitro phenotypic characterization through multiple biological assays, firstly to verify the neoplastic nature of the MF-R 3 cell line and secondly to investigate the validity of MF-R 3 2D and 3D cultures as preclinical models to recapitulate the morphologic and phenotypic characteristics of the original tumour, increasing the spectrum of available tools to be used in different experimental settings.

## Materials and methods

### Patient’s history

**Fig. 1:**
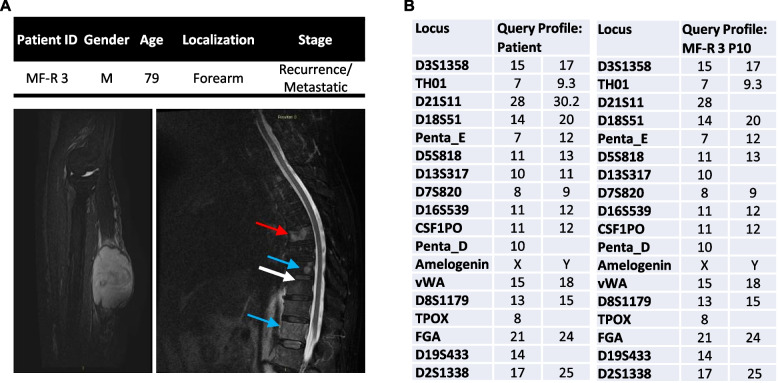
Patient’s data and generation of MFS derived cell line. **A** Summary of clinical data of the patient (top), Sag T2 FAT Sat MRI of the forearm shows the recurred tumour mass (bottom left), while Sag T2 FAT Sat MRI of the spine (bottom right) shows multiple hyperintense metastatic lesions (blue arrows) with a pathological fracture in T6 (red arrow). **B** STR profiling data obtained by ATCC of the patient’s tumour sample (left) and MF-R 3 cells at passage 10 (hypothesized immortalization passage) (right)

The MFS cell line, named MF-R 3, was obtained from a 79-year-old man, previously diagnosed with high-grade MFS in another hospital, who underwent surgery at the Istituto Ortopedico Rizzoli for a grade 3 MFS second recurrence, localized at the left forearm (see MRI images, Fig. 1A). In the following months the patient presented vertebral metastases (see MRI Fig. 1A bottom right) and died a few months later. Histological diagnosis of MFS was performed by experienced sarcoma pathologists.

The research protocol was approved by the Local Ethics Committees (AVEC and CEROM). The number of Ethical Committee approval is PG N. 0012825 of 12/11/2018.

### Cell line isolation, culture, and authentication

The MF-R 3 cell line was obtained by mechanical fragmentation and enzymatic digestion of patient tumoural samples as described in De Vita *et al.* [19] with minor modifications. Specifically, the sample was treated with 2 mg/ml collagenase type I-A (Sigma-Aldrich, Merck Group) in D-PBS with Ca^2+^ Mg^2+^ (Life-Technologies) for 2 hours at 37°C in a humidified incubator. A total of 4 ml of this digestion buffer were used to digest 1 g of fresh tumour tissue. The obtained suspension was filtered with a 100 µm cell strainer, centrifuged for 10 min at 460 g, resuspended in fresh medium, and counted. Cells were seeded at 8*10^4^ cells/cm^2^ density and then passaged once a week at 2*10^3^ cells/cm^2^ density.

The 143B human osteosarcoma cell line was obtained by ATCC and was chosen as control in each experiment as an example of an extensively studied and characterised aggressive sarcoma cell line, unless differently specified.

All cell lines were cultured in DMEM high glucose supplemented with 10% lot-selected FBS, 1% L-glutamine, and 1% Penicillin/Streptomycin (all from Life Technologies). For the MFS cell line, amphotericin B (Lonza) was added to the medium in the first passages. All cells were cultured in a monolayer at 37 °C and 5% CO_2_ in a humidified incubator and were mycoplasma-free.

Cell line authentication was performed by ATCC through STR analyses (Cell Line Authentication kit, ATCC); MF-R 3 cells at passage 10 and cells obtained from another portion of the tumoural tissue present in the Department of Pathology and stored at the Musculoskeletal Tumor Biobank (BIOTUM) of the Istituto Ortopedico Rizzoli were sent to ATCC.

All the experiments have been conducted after passage 13, when the number of population doublings after the first passage offered a reliable first sign of MF-R 3 cells’ spontaneous immortalization.

### Cellular proliferation assay

MF-R 3 and 143B cells were seeded at 2*10^3^ cells/cm^2^ density in technical quintuplicate into 96-well plates and cultured for 7 days at standard conditions. At each time point (days 1, 2, 3, 4, and 7 after seeding), cells were fixed with 10% neutral buffered formalin (Diapath S.p.A) for 30 minutes at RT and stained for 30 minutes in the dark with 100 µl of 1% methylene blue solution in 0.01 M borate buffer pH 8.5. Cells were then washed three times with 0.01 M borate buffer and methylene blue was dissolved with 100 µl per well of ethanol 100%: HCl 0.1 M 1:1 (v/v) ratio for 3-5 minutes in the dark in agitation. When the staining solution appeared clearly dissolved, absorbance was read at λ=655 nm using a microplate reader with an empty plate as background signal (Synergy HT, Bio-Tek Instruments Inc.). Cell number was calculated from blank corrected absorbance interpolated against a reference standard curve.

### Two-dimensional clonogenic assay

For each cell line, 100 cells/well in a 6-well plate were seeded and the colony number was evaluated after 7 days for 143B and after 10-14 days for the MF-R 3 cell line. Cells were fixed overnight in 70% Ethanol and stained for 15 minutes with 1% methylene blue solution in 0.01 M borate buffer pH 8.5. Colonies were then washed 3 times in diH_2_O and manually counted. Clonal efficiency was calculated as the percent ratio between the average number of counted colonies and the number of seeded cells.

### Selective nucleolar silver-staining (AgNOR)

For the selective silver-staining of nucleoli, a confluent T150 flask of cells was trypsinized, pelleted, and then fixed overnight at 4°C in 10% neutral buffered formalin (Diapath S.p.A). The day after, cell pellets were washed three times with PBS and then resuspended in 100 µl of 1% low melting agarose (Lonza). The suspension was then poured into disposable cryomolds 7x7x5 mm and incubated at 4°C for at least 10 minutes to allow agar solidification. Once solid, samples were moved to histological cassettes for paraffin inclusion. For each sample, 5 µm-sections were deparaffinized and re-hydrated. The antigen retrieval step was performed in autoclave in citrate buffer at pH 6. Silver staining was carried out following the “one step” procedure described by Ploton *et al.* [27]; in brief, slices from the cell pellets were incubated for 13 min at 37°C in silver nitrate solution at 30% (w/v) in 2% gelatin, 1% aqueous formic acid. The slices were then washed several times in water, dehydrated, and mounted with coverslips. For each slide, 5 images were captured using a Diaplan Leitz microscope with a 40X objective, and nucleolar size was measured on an average of 20 cells per field using a custom-made macro on ImagePro Plus Analyser software. Quantification analyses were performed according to the guidelines of the “International Committee on AgNOR Quantitation” [28].

### In Vitro wound healing assay

Cells were seeded at a density of 2.5*10^4^/cm^2^ in a 6-well plate and grown until 100% confluence. The cell monolayer was wounded with a p200 pipette tip, washed with PBS, and kept in 2 ml of fresh medium. To monitor the wound closure, pictures were taken at 0, 16, 20, 24, 40, and 44 hours after the scratch at 40X magnification through an inverted Nikon Eclipse TE2000-U microscope equipped with a calibrated Nikon DS-Vi1- U3 CCD camera. The areas of wound closure were calculated with the Area auto-detection tool of NIS Elements D software (Nikon) on the captured images.

### Invasion assay

Cell invasive potential was tested by seeding 2,5*10^4^ cells in 1% FBS culture medium on the upper chamber of a PET transwell insert with 8 µm pores (Merck Group) in a 24-well plate. To simulate the presence of surrounding tissue, the upper side of the insert was previously coated with a thin layer of Type I collagen from rat tail tendon (Roche; 2 mg/ml in 0.1 % acetic acid, 8:1:1 v/v collagen:HEPES 10X:DMEM 10X to buffer pH to 7). Non-coated transwell control was performed for each experiment. Complete medium with 10% FBS was added in the lower compartment of each well to generate a chemotactic stimulus. The staining procedure has been performed as described in Galbiati *et al.* [29]. The number of invading cells was obtained by counting five random fields of each insert, at 100X magnification using an inverted Nikon Eclipse TE2000-U microscope.

### Soft agar colony formation (SACF) assay

SACF assay has been performed as described by Kusakawa *et al*. [30], with minor adjustments. In brief, 24-well plates were used to dispense 500 µl per well of agar 0.5% [1:1 v/v Low Melting Agarose (NuSieve™ GTG™, Lonza) 1% in D-PBS:DMEM - Low glucose 2x (powder, Sigma Aldrich)] as the bottom agar portion. During the agar solidification step of 30 minutes at 4°C, cells were trypsinized, counted, and seeded as single cell suspension as follows: 2.5*10^3^ cells/well were carefully suspended in 100 µl of DMEM - Low glucose 2x, then 50 µl of melted agar 1% in PBS was gently added to cells suspension. Immediately after, 150 µl of the mixture was dispensed in every single well. The agar solidification was allowed for 15 minutes at 4°C. Subsequently, 1 ml of fresh culture medium was added to each well and plates were placed in the incubator for 14 days. Six hundred microliters of fresh medium were replaced every 3/4 days, without disturbing the cells in the agar layer. After 2 weeks, colonies were counted under an inverted Nikon Eclipse TE2000-U microscope at 40X magnification using a home-made grid as track. Colony-forming efficiency has been calculated as in Kusakawa *et al*. [30].

### Low-density anchorage-independent growth of three-dimensional aggregates (myxospheres)

For 3D aggregate culture, cells were seeded at 600 cell/ml density in ultra-low attachment 6-well plates (Corning inc) in 2 ml of complete medium. Three-dimensional aggregates started to be visible to the naked eye after 1 week and passaged every 2 weeks. For passaging, aggregates were centrifuged and resuspended in 500 µl of fresh medium, then a single-cell suspension was obtained by pipetting the pellet with a 1 ml syringe with a 26G needle (BD Franklin Lakes). Erythrosine B stain was used to discriminate and count viable cells through Countess™ II FL Cell Counter (Life Technologies).

### Transmission Electron Microscopy (TEM) analysis

For ultrastructural evaluation, MF-R 3 cells were fixed with 2.5% glutaraldehyde in 0.1 M cacodylate buffer pH 7.4 for 1 h at room temperature. Afterwards, samples were postfixed with 1% osmium tetroxide in 0.1 M cacodylate buffer for 1 h at 4°C, dehydrated in an ethanol series, infiltrated with propylene oxide, and embedded in Epon resin. Ultrathin sections (80 nm thick) were stained with uranyl acetate and lead citrate (15 min each) and observed with a Jeol Jem 1011 transmission electron microscope operated at 100 kV. Images were captured using an Olympus digital camera and iTEM software.

### Seeding of MF-R 3 on the chorioallantoic membrane (CAM)

Fertilized eggs were purchased from the Chick Farm company (Faenza, Italy) on day 1 post-fertilization and kept at 37°C with 56% humidity with scheduled rotation. On day 4 post-fertilization all unfertilized eggs were discarded. On day 5 post-fertilization, 2-3 ml of albumen were removed, and a window was opened on the eggs’ shell. To avoid contamination and damage to the embryo, the window was kept closed with a piece of parafilm and opened only in case of direct intervention or imaging. On day 8 post-fertilization, 4*10^5^ MF-R 3 cells were mixed with LDEV Reduced Growth Factor Geltrex (1:1 v/v, 50 µl total final volume; Gibco™ Life Technologies) and seeded on the CAM. On day 15 post-fertilization, the chick embryos were euthanized, and the tumour masses were removed for further processing. A well distinguishable neoformation was visible in all the CAMs. Tissue samples were placed in the histological cassette and fixed in formalin (Merck Millipore) for at least 2 hours at +4°C and paraffin embedded.

### Histology

4 µm thick histological sections were obtained by formalin-fixed, paraffin-embedded tumoural samples and stained with hematoxylin & eosin in an automatic slides stainer.

### 2D and 3D drug sensitivity assay

For 2D drug sensitivity tests, for both cell lines, 5*10^3^ cells/well were seeded in black 96-well clear-bottom plates. After 24 hours, cells were treated with a series of 10-fold dilution of Epirubicin (Sigma-Aldrich, Merck Group) or Doxorubicin (Sigma Aldrich, Merck Group) in complete medium with final concentration ranging from 100 µM to 0 µM. Both drugs were resuspended in DMSO following the manufacturer’s instructions. In each experiment the maximum concentration of DMSO was tested for potential toxicity (0.1% for Epirubicin and 0.2% for Doxorubicin). Cell viability was assessed 72 hours after treatment using PrestoBlue™ (Invitrogen) diluted at 10% in complete medium and incubated for 2 hours at 37°C and 5% CO_2_ in a humidified incubator. Fluorescence measurement (ex 530 nm/em 590 nm) was performed using a microplate reader (Synergy HT, Bio-Tek Instruments Inc.).

For 3D drug sensitivity tests, both cell lines had 500 cells/well seeded in black Ultra-low Attachment 96-well plates with clear round bottom. Before treatment, spheroids’ formation was allowed for 5 days. Drug treatments were performed as described for 2D tests. Spheroids viability was assessed using CellTiter-Glo® 3D (Promega) following manufacturer’s protocol. Luminescence was measured using a microplate reader (Synergy HT, Bio-Tek Instruments Inc.).

### Statistical analyses

The data have been analysed using GraphPad Prism 8.4.3. For each experiment, at least three biological and technical replicates were performed. Values of *p* lower than 0.05 were regarded as statistically significant.

## Results

### Establishment and characterization of the MFS patient-derived cell line

MFS patient-derived cell line was obtained after mechanical and enzymatic digestion of selected portions of the tumour mass as described in the Materials and Methods section (Fig. 1A, B). Cell line authentication through STR profiling confirmed the correspondence to the patient’s biological material (Fig. 1B), and the absence of similarities with already existing cell lines in the ATCC database. Interestingly, loss of heterozygosity (LOH) phenomena have been detected in MF-R 3 cells.

**Fig. 2:**
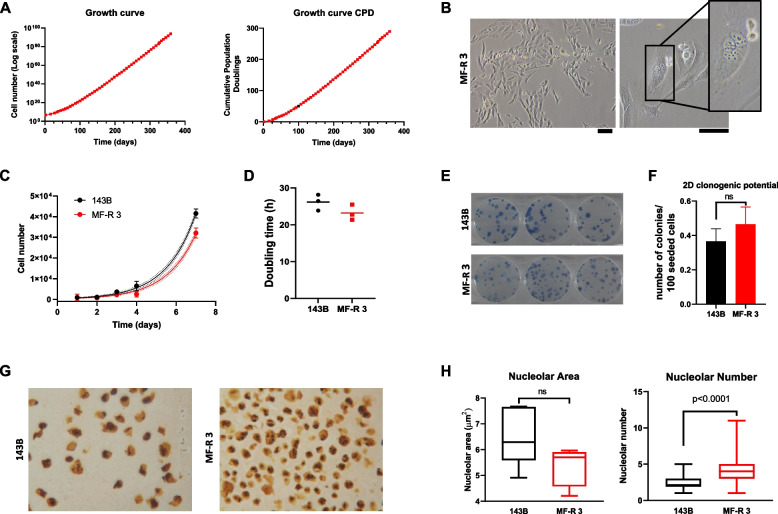
Biological characterization of the MF-R 3 cell line. **A** Growth curve of MF-R 3 expressed as cell number (left) and cumulative population doublings (CPD, right). CPD were calculated at each passage as log2(N1/N0), where N0 is the number of cells seeded and N1 is the number of cells harvested at the end of the passage. **B** Representative images of morphological aspects of MFS cells after spontaneous immortalization. Scale bar = 100 µm. **C** Representative result of proliferation assay: dots and error bars represent the cell number at the indicated time point (mean ± SD of 5 technical replicates), continuous and dotted lines indicate the interpolated exponential growth curves with 95% confidence intervals. **D** Doubling time of MF-R 3 and 143B cell lines. Graph shows single DTs (dots) calculated from exponential growth equations (example shown in C), with mean (lines). **E** Representative images of colony forming assay. **F** Clonogenic potential in 2D: 100 cells/well were seeded in a 6-well plate and Methylene blue stained colonies were counted after 7 (143B) or 14 days (MF-R 3). **G** Representative images of AgNOR staining of 143B (left) and MF-R 3 (right). **H** Average nucleolar area above 4 µm.^2^ (left) and number of nucleoli (right)

MF-R 3 cells were grown for more than 350 days in culture, reaching almost 290 population doublings (PD) (Fig. 2A). They are a fast-growing cell line, composed mostly of small spindle-shaped cells, with few larger multinucleated cells, likely due to events of aberrant mitosis. Interestingly, several semi-floating cells are always present alongside the prominent population of adherent cells (Fig. 2B).

We found a similar growth rate of MF-R 3 cells compared to the osteosarcoma cell line 143B, used as a reference of an aggressive sarcoma cell line (Fig. 2C), and a slightly lower doubling time (DT) of MFS cells (21±2.1 h for MF-R 3) than 143B cells’ DT (24±2.2 h) (Fig. 2D). Furthermore, when seeded at very low-density MFS cell line can grow and form colonies, similarly to 143B cells (Fig. 2E,F).

Ribosome biogenesis is a well-known indicator of cellular replication [31]. As a readout of ribosome biogenesis activity, we performed selective nucleolar silver staining (AgNOR, Fig. 2G) and we measured nucleolar area, which has previously been correlated to cell DT and cancer growth rate (Fig. 2H left) [32, 33]. Notably, the number of nucleoli is higher in MF-R 3 cells than in 143B cells (Fig. 2G and H right panel), even though their average total area is lower, around 5 µm^2^ (Fig. 2H left).

### MF-R 3 cells show remarkable migration and invasion potentials

**Fig. 3:**
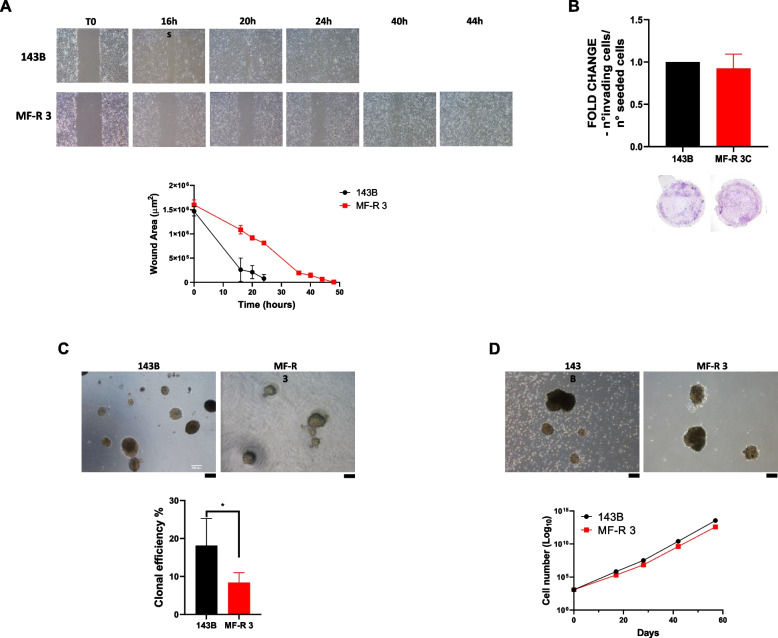
MF-R 3 cell line shows an aggressive phenotype in vitro. **A** Wound healing assay: representative images of cells closing the scratch (top); Graph reporting the area of the plate not covered by cells (wound area) at the different timepoints (bottom); **B** Invasive potential assay through 24-well transwell filter coated with collagen. For each filter sample, five random fields were counted. Graph shows the ratio between the number of invading cells and the number of seeded cells compared to 143B results (fold change). Representative pictures of transwell filters are shown below the graph; **C** Soft agar colony formation assay: representative images of colonies formed by single cells embedded in 0,33% low-melting agar after 2 weeks of culture (top) and clonal efficiency (bottom); **D** Anchorage-independent propagation of MF-R 3 and 143B: representative images of cell aggregates formed in ultra-low attachment plates (top) and proliferation of cells maintained in non-adherent culture (bottom)

Since one of the hallmarks of cancer cells and one of the main features of MFS cells is to invade the surrounding tissues, we performed a wound healing assay. Results demonstrate that these cells can close the gap in 44 hours, and even if the MF-R 3 wound closure rate is inferior compared to 143B cells it is still remarkable (Fig. 3A). Moreover, MF-R 3 cells can migrate through a collagen-coated transwell membrane showing strong invasive potential, notably reaching similar results to 143B cells (Fig. 3B).

### MF-R 3 cells form 3D colonies in anchorage-independent conditions and grow as 3D cellular aggregates

As further index of neoplastic behaviour and aggressiveness, we studied the ability of anchorage-independent colony growth by the SACF assay. Results indicate that MF-R 3 cell line has high SACF efficiency even if it’s lower compared to the one observed for 143B cells (Fig. 3C). To further investigating the aggressive character of MF-R 3 cells and uncovering possible signs of stemness, we challenged them to grow in low-density free-floating conditions using ultra-low attachment plates. The MFS cell line show sustained growth in 3D aggregates, that we named myxospheres, that can also be propagated in culture (Fig. 3D). As expected, as myxospheres the cells grow at a lower rate than the 2D culture. In fact, in the 2D proliferation assay, we observed an exponential growth with 5 PD in one week (Fig. 2A right), while in the myxospheres the number of cells retrieved after 17 days accounted for an equivalent of 6 PD (Fig. 3D bottom).

### MF-R 3 cells are useful for the generation of near-patient preclinical models

**Fig. 4:**
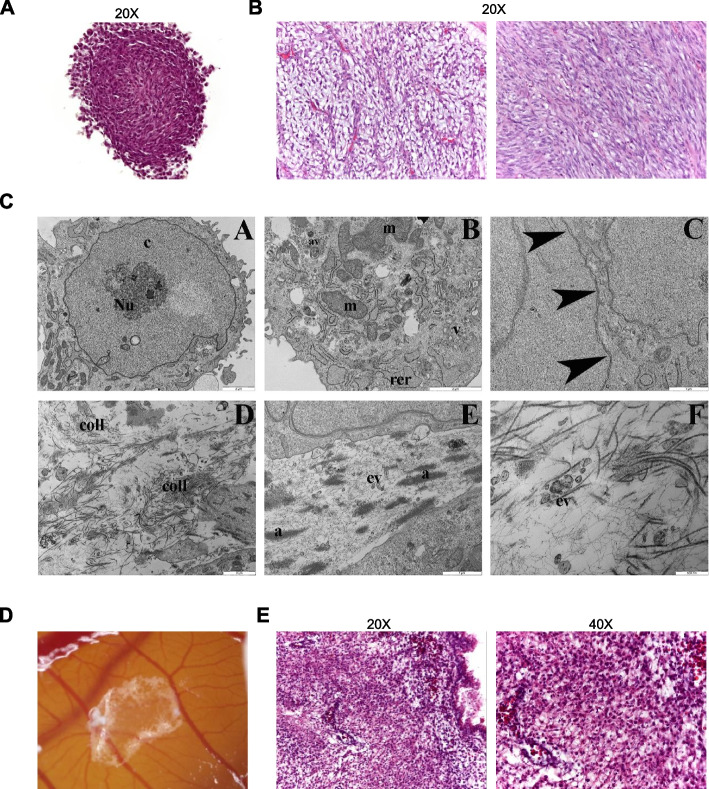
MF-R 3 cell line is useful to generate pathologically relevant preclinical models. **A** Representative image of hematoxylin and eosin staining of one myxosphere originating from MF-R 3 cells seeded in ultra-low attachment conditions at very low cellular density. Magnification 20X. **B** Pictures of the hematoxylin and eosin staining of patient’s tumour: myxoid area (left) and high cellularity area (right). Magnification 20X. **C** Panel of six representative pictures of TEM analysed samples. Interesting features are described in the main text. **D** Representative picture of the tumour mass generated by MF-R 3 cells grown on CAM. **E** Representative images of hematoxylin and eosin staining of the tumour mass generated by MF-R 3 cells seeded on CAM of chick embryos. Magnification 20X (left) and 40X (right)

To verify if MF-R 3 cell line could be useful to generate pathologically relevant preclinical models as tumorspheres [34], we characterised the morphology and ultrastructural features of the myxospheres through immunohistochemical and electron microscopy analyses. Haematoxylin and eosin staining show that MF-R 3 cells growing as myxospheres completely recreate the morphology and architecture of the areas of high cellularity of the patient’s tumour mass (Fig. 4A and B).

By TEM, MF-R 3 myxospheres are characterised by large cells with high nuclear-cytoplasmic ratios and relatively smooth surfaces. The large nuclei have an irregular shape with dispersed chromatin (c) and prominent nucleoli (Nu) as demonstrated by AgNOR staining (Fig. 4C, picture A). In the cytoplasm, we observed mitochondria (m) that varied in size and shape with dense matrix and differently oriented cristae, but most of them tended to be oval or elongated (Fig. 4C, picture B). Abundant rough endoplasmic reticulum composed of long, dilated, and thin cisternae are also present together with multivesicular bodies, autophagic vacuoles (av), and numerous endosomes and vesicles (v) indicating cytoplasmic endocytotic and exocytotic activities (Fig. 4C, picture B). A few lipid droplets were noted. In addition, cell bodies are closely flacked without the presence of evident intercellular junctions suggesting a cell ability to detach from the 3D mass (black arrowheads) (Fig. 4C, picture C). Unexpectedly, we noted the presence of abundant deposition of banded collagen fibrils (coll) distributed randomly and not orientated throughout the extracellular space (Fig. 4C, picture D) as well as aggregates of collagenous filaments (a) together to numerous extracellular vesicles (ev) that could be ascribable to matrix vesicles suggesting an initial matrix mineralization (Fig. 4C, pictures E and F). In parallel, we seeded MF-R 3 cells on the CAMs of chick embryos with the aim to challenge the cells to grow in a complex viable system. This cell line formed tumour masses with high similarity to the patient’s one (Fig. 4D and E).

### MF-R 3 cell line has a good response to anthracyclines both in 2D and 3D drugs screening tests.

**Fig. 5:**
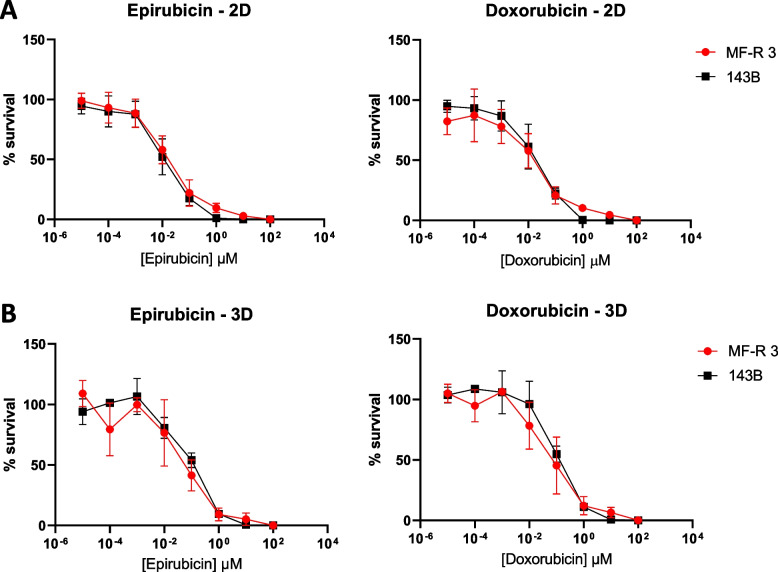
MF-R 3 cells show sensitivity to anthracyclines-based therapy both in 2D and 3D. **A** Percentage of survival curves after 72 h of treatment with logarithmic increasing doses of epirubicin (left) and doxorubicin (right) in 2D cell culture settings. **B** Percentage of survival curves after 72 h of treatment with logarithmic increasing doses of epirubicin (left) and doxorubicin (right) in 3D cellular spheroids

We performed anthracycline-sensitivity tests on 2D and 3D cultures (cellular spheroids). Data obtained from 2D tests show that MF-R 3 cells have similar sensitivity to that of 143B cell line for both drugs (Fig. 5A). Interestingly, when challenged in a 3D culture configuration, both cell lines resist higher doses of both drugs. In addition, a trend of a higher effect on MFS-derived cells is visible, reflecting the histopathological differences between the two cell lines (Fig. 5B).

## Discussion

MFS is characterised by an extreme cellular and molecular heterogeneity. To unravel the complex molecular pathogenesis of this sarcoma and to investigate the validity and mode of action of standard and innovative therapies, there is an urgent need to expand the availability of preclinical models. In this work, we present a novel spontaneously immortalised MFS-patient-derived cell line, named MF-R 3, that recapitulates in 2D and 3D clinically relevant features of the original tumour. When grown in traditional monolayer culture, MF-R 3 cell line is composed prevalently of small spindle-shaped cells as most of the published MFS cell lines [21, 22, 35–38]. A small fraction of the cells shows the tendency to not completely adhere to plastic (Fig. 2B). Notably, as other research groups noticed, we reported the presence of large, rare multinucleated cells [5, 12, 20, 39]. STR analyses confirmed the genetic correspondence between the patient’s tumour sample and MF-R 3 cells, together with the isolation of a new cell line that is absent in the ATCC database. STR analyses also pointed out LOH phenomena at passage 10 (Fig. 1C). LOH is common in sarcoma cell lines cultivated in vitro and also occurred in other published MFS cell lines, even though the passage at which the analysis has been conducted is not always publicly available [35–38]. Successively we proceeded with the evaluation of cellular proliferation and DT, finding a continuous and fast growth of our MFS cell line with a DT of 21 hours, only slightly shorter than that of the well-known aggressive osteosarcoma cell line 143B (Fig. 2C and D).

The choice to use the osteosarcoma cell line 143B as reference to compare this new MFS cell line was dictated by three major points: i) each of soft tissue sarcomas (STSs) subtype has distinct features that make it unique and difficult to compare with other STSs; ii) 143B cell line has been extensively studied and characterised in literature; iii) 143B cells are particularly aggressive due to their metastatic nature. Moreover, a similar decision was taken by Professor Kondo’s group to assess the migration of their myxofibrosarcoma cell lines NCC-MFS4-C1 and NCC-MFS6-C1, in which the used an OS cell as reference [36, 38]. The unexpectedly short DT of MF-R 3 can be explained by the cell line originating from a second recurrence, since cell growth characteristics are likely related to the clinical stage and tumour grade of the original biological material. DT reported for other MFS cell lines are higher except for the NCC-MFS2-C1 (13 h) [22] and MUG-Myx1 (24 h) [7] cell lines. Kondo’s group suggested that long DT fit with the slow growth of MFS in vivo [21, 22]*.* Unfortunately, clinical data are not fully disclosed for these cell lines, curtailing further speculation.

MF-R 3 cells grown in monolayer display multiple large nucleoli (Fig. 2B), as described by Miserocchi *et al.* in the IM-MFS-1 cell line [6], and by Lohberger *et al.* in the MUGMyx1 cell line [7]. The nucleolus is the area where ribosome biogenesis, namely the process of ribosome production, takes place in eukaryotic cells. Ribosome biogenesis is directly proportional to protein demand, which is usually higher in cancer cells than in normal cells to maintain an increased proliferation rate. Furthermore, nucleolar irregularities in size and shape have been correlated to cancer and are recognized as valuable biomarkers of cancerogenesis and aggressiveness in several human tumours [31–33].

TEM images in Fig. 4C show, for the first time, the ultrastructural details of a primary MFS cell culture, enlightening the irregular and hypertrophic shape of MF-R 3 nucleoli. We also performed, for the first time on MFS samples, AgNOR staining and evaluated the number and the size of nucleoli (Fig. 2F and G). MF-R 3 cells have more nucleoli than 143B cells (Fig. 2G right), affirming their aggressive neoplastic behaviour. On the other hand, MF-R 3 nucleolar area is smaller than that of 143B (Fig. 2G left), probably due to the intrinsic differences between myxofibrosarcoma and osteosarcoma. Interestingly, it has been demonstrated an inverse correlation between the nucleolar area and the DT [32], thus being in contrast with our data that however are not statistically significant. This may be ascribed to fundamental differences respect to carcinomas and other human tumours as it has been observed for many other morphological, clinical, and therapeutic aspects. To the best of our knowledge, few studies on the role of ribosome biogenesis in sarcomas have been conducted encouraging a deeper investigation of its possible role in myxofibrosarcoma pathogenesis. Results obtained from wound healing and invasion assays showed the good migratory abilities of MF-R 3 cells (Fig. 3A) and importantly, their ability to penetrate a collagen layer, similar to the highly invasive cell line 143B [40]. This great in vitro invasive potential reflects the highly invasive and metastatic nature of the tumour of origin indicated by the presence of vertebral metastases. Both migratory and invasive potentials have also been evaluated for some other MFS cell lines; the overall data reflect the high infiltrative nature of MFS that is well recreated by the majority of MFS cell lines [12, 21, 22, 35–38].

MFS is characterised by multinodular growth, particularly when superficially located as in the case of this patient [1, 41]. Interestingly, MF-R 3 were able to form 2D colonies similarly to 143B cells (Fig. 2F), grew and formed 3D colonies in soft agar indicating a strong neoplastic and staminal potential [30, 42]. Even if the clonal efficiency is lower than that of 143B cells, our result is in line with other publications on either continuous cancer cell lines or primary cell cultures [30, 43–45]. The results of 2D and 3D clonogenic potentials indicate a staminal nature and the ability to replicate and form a growing mass even from a very low cell number and in challenging conditions. These observations concord with the tendency of MFS to recur in the same anatomical site, especially in the case of surgery with positive margins when few residual cells may be still viable [14, 18, 46]. Of note, MF-R 3 cells were isolated from a second local recurrence.

Interestingly, MF-R 3 cells can grow and be propagated in suspension as 3D cellular aggregates, named myxospheres (Fig. 3D), that completely recreate the high cellularity areas of the patient tumour mass (Fig. 4A and B). Other primary MFS cell lines demonstrated the ability to grow forming aggregates of highly proliferating atypical cells, although seeding conditions and culture settings were different [21, 35, 38]. TEM analyses on MF-R 3 -derived myxospheres clearly show high-dense euchromatin areas and hypertrophic nucleoli, both signs of great proliferation and productive cellular activity (Fig. 4C, picture A). Additionally, the presence of abundant and large rough endoplasmic reticulum, multivesicular bodies, autophagic vacuoles (av), and numerous endosomes and vesicles (v) are further signals of great endocytotic and exocytotic activities, which indicate high cell-to-cell communication (Fig. 4C, picture B). The exchange of this information seems to be mainly carried out through vesicle trafficking instead of other membrane transport mechanisms. In fact, MF-R 3 cells inside the myxospheres grow very close to each other but without structured cellular junctions (Fig. 4C, picture C), in line with the high migratory and invasive potentials of these cells. The presence of lipid droplets observed in the cytoplasm by TEM analyses together with altered and numerous mitochondria may suggest a dysregulation in fatty acid metabolism which has been frequently associated with cancer [47]. Up to now, no wet- or dry-lab research has been published on the role of fatty acid metabolism in MFS and worth further investigation. Surprisingly, TEM images reveal the production of ECM made by randomly orientated banded collagen fibrils (Fig. 4C, picture D) that, in some regions, are aggregated together with numerous vesicles (Fig. 4C, pictures E and F) suggesting the presence of matrix vesicles and an initial stage of matrix mineralization. We know that the tumour of origin infiltrated the adjacent bone tissue, and we can speculate that this contact might have influenced the ECM production program of the tumour cells. Further studies would be necessary to fully elucidate this aspect.

Finally, we evaluated both in 2D and 3D the efficacy of anthracycline-based therapy, specifically doxorubicin and epirubicin, that are usually administered as a palliative treatment prior to or after surgery [15] (Fig. 5). In this experiment we used spheroids as a 3D model to have a more controlled system with respect to myxospheres in terms of cell number, shape, and dimension of the 3D structure. Results show that 3D spheroids of both cell lines are more resistant to anthracyclines treatment than standard 2D cultures (Fig. 5). More interestingly, MF-R 3 spheroids are slightly more sensitive than 143B spheroids, probably because of the diverse tumour histotype (Fig. 5B). In addition, the myxoid matrix produced by MFS cells facilitates the diffusion of nutrients and other molecules [48], thus probably allowing a better diffusion of drugs inside the spheroid. Similarly, Tsuchiya *et al.* tested cell viability after exposure to different concentrations of doxorubicin in five MFS-derived cell lines [37]. Compared to these cell lines, MF-R 3 cells are strongly more sensitive to doxorubicin treatment probably due to the major effects of anthracyclines on rapidly proliferating cells. In fact, the NCC-MFS2-C1 cell line, which has a DT of 13 hours, has a similar sensitivity to gemcitabine and topotecan, both drugs having a comparable mechanism of action to doxorubicin. Additionally, Kito *et al.* tested the efficacy of mitoxantrone, an anthracenedione antineoplastic agent that completely shares the mechanism of action with the anthracyclines we selected. Also in this case, NCC-MFS1-C1 cell line has a more resistant phenotype to this drug than that of MF-R 3 to doxorubicin and epirubicin. Notably, NCC-MFS1-C1’s DT is very long compared to that of MF-R 3 cells (78 h vs 21 h) [21].

Our results demonstrate that we successfully isolated an aggressive MFS-derived cell line that recapitulates the pathologically relevant features of the patient’s tumour mass. This cell line joins the group of the other preclinical tools available to study the mechanisms that may have a role in the pathogenesis of MFS, including ribosome biogenesis and fatty acid metabolism.

## Conclusions

In the present paper, we successfully isolated and characterised a novel MFS-derived cell line that shows aggressive phenotype resembling the patient’s clinical behaviour. MF-R 3 cells are also a suitable tool to generate diverse 2D and 3D near-patient preclinical models that may be useful to deepen the knowledge of MFS pathogenesis and drugs response. For the first time in literature, we also present nucleolar staining and TEM analyses on our cell line, that suggest us to expand the research on new biological pathways, such as ribosome biogenesis and mitochondrial fatty acid metabolism, that may hide some interesting answers for MFS management.

## Data Availability

All data generated or analysed during this study are included in this published article and are available from the corresponding author upon reasonable request.

## Abbreviations

CAM: Chorioallantoic Membrane
CPD: Cumulative Population Doublings
DT: Doubling Time
ECM: Extra Cellular Matrix
LOH: Loss Of Heterozygosity
MALDI: Matrix Assisted Laser Desorption/Ionization
MFS: Myxofibrosarcoma
MSI: Mass Spectrometry Imaging
PD: Population doublings
PDOXs: Patient-derived Orthotopic Xenografts
PDXs: Patient-derived Xenografts
SACF: Soft agar colony formation
STS: Soft Tissue Sarcoma
TEM: Transmission Electron Microscopy
